# Reduced ejection fraction as the sole potential limitation for wire pacing in transfemoral aortic valve replacement

**DOI:** 10.3389/fcvm.2025.1579898

**Published:** 2026-02-18

**Authors:** Sascha d'Almeida, Tilman Stephan, Susanna Jörk, Stefanie Andreß, Marvin Krohn-Grimberghe, Johannes Mörike, Wolfgang Rottbauer, Birgid Gonska, Dominik Buckert

**Affiliations:** Department of Cardiology, Angiology, Pneumology and Internal Intensive Care, University of Ulm, Ulm, Germany

**Keywords:** LVEF/left ventricular ejection fraction, rapid pacing, TAVI, TAVR, transvenous pacemaker, wire pacing

## Abstract

**Background:**

In recent years, fast-track protocols have been developed for transcatheter aortic valve replacement/implantation (TAVR/TAVI) to further simplify the procedure. Traditionally, intraprocedural rapid ventricular pacing for valve deployment is achieved via a transvenous temporary pacing wire inserted into the right ventricle.

**Aim:**

The aim of the present study was to assess the safety and efficacy of a temporary left ventricular pacing (wire pacing) technique during the TAVI procedure.

**Methods:**

In this prospective observational study, 307 consecutive patients undergoing TAVI with either the Edwards SAPIEN S3 (*N* = 161; 52.4%) or the Medtronic Evolut Pro+ (*N* = 146; 47.6%) were included. Left ventricular pacing was performed using the valve delivery guidewire. The primary endpoint was defined as successful rapid pacing. Safety outcomes and both qualitative and quantitative secondary endpoints were also analyzed and compared to those of a conventional previous TAVI cohort that received transvenous right ventricular pacing.

**Results:**

Left ventricular pacing was successful in 93.8% of cases (*n* = 288). Moreover, 16 patients (5.4%) required a temporary pacing wire during the procedure due to complete heart block. Procedural success of TAVI was achieved in 98.0% of patients (*n* = 294). Periprocedural complications were low and included an in-hospital death rate of 1.6%, major bleeding in 1.3% of patients, and permanent pacemaker implantation in 10.2% of patients following TAVI. No incidence of cardiac perforation or tamponade was observed. Safety outcomes were comparable to those of the conventional right ventricular pacing group. Reduced left ventricular ejection fraction (LVEF) was significantly associated with unsuccessful left ventricular guidewire pacing (*p* = 0.036). An LVEF cutoff of <52% best predicted left ventricular stimulation failure (area under the curve 0.632, sensitivity 63.2%, specificity 63.8%, *p* = 0.045).

**Conclusion:**

Wire pacing is safe and effective, should be preferred for TAVI, and eliminates the risk of complications associated with transvenous pacing wires. Nonetheless, caution is advised when using this technique in patients with reduced ejection fraction or a high risk of conduction disorders.

## Graphical Abstract

**Figure:**
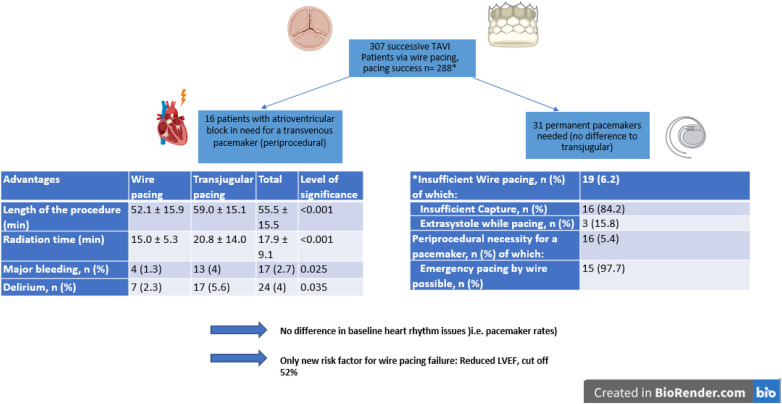
Advantages and limitations of wire pacing in TAVI.

## Introduction

Transcatheter aortic valve implantation (TAVI) has become a cornerstone in the treatment of severe aortic valve stenosis, offering an effective and minimally invasive alternative to surgical aortic valve replacement. Within the last 15 years, the number of implanted valves has continued to increase gradually (1), accompanied by steady improvements in many aspects of periprocedural management. Advances in TAVI techniques have focused on simplifying the procedure and minimizing complications, particularly through the implementation of fast-track protocols aimed at improving procedural efficiency and patient recovery. One critical aspect of the procedure is rapid ventricular pacing, which temporarily reduces cardiac output to facilitate precise valve deployment. Traditionally, this is achieved by inserting a transvenous pacing wire into the right ventricle (2). However, this approach comes with additional radiation exposure time and carries inherent risks, including mechanical complications such as perforation, tricuspid valve damage, and pericardial tamponade (3, 4).

To prevent these complications, alternative pacing strategies have been explored, including left ventricular pacing via the valve delivery guidewire. This novel approach eliminates the need for an additional pacing wire, potentially reducing procedural complexity and associated risks. Several smaller studies have created cohorts attempting to implant TAVI by pacing the patient via an arterial wire (5–7) and have validated the effectiveness of this method (6, 7). The present study evaluates the safety and efficacy of this technique in a large group of TAVI patients, with a particular focus on procedural success, periprocedural complications, and patient-specific predictors of left ventricular pacing failure.

By comparing this method with conventional right ventricular pacing, this study aims to provide evidence for a streamlined intraprocedural pacing approach that may become the new standard during TAVI, while also defining cases in which traditional transjugular pacing should be preferred or kept on standby.

## Methods and study population

Among patients with symptomatic severe aortic stenosis planned for a TAVI procedure at our heart center in the University Heart Center in Ulm, Germany, we consecutively included 307 patients who all received a transfemoral wire pacing starting from 9 April 2022. None of the patients were excluded. Wire pacing was performed as previously described by Faurie et al. in 2016 (6, 7). Briefly, the tip of the back-up 0.035″ guidewire was connected to the cathode of an external pacemaker, while the anode was attached to a needle inserted into the groin. The inserted TAVI or balloon catheter provided the necessary isolation. Successful wire pacing was defined as completion of a successful TAVI procedure with the need for a transjugular pacemaker. This included wire pacing for TAVI implantation as well as wire pacing as a transient treatment in cases of relevant conductance disturbances. For Evolut valves, Safari and Lunderquist extra-stiff guidewires were used, whereas Edwards valves were implanted using Amplatzer extra-stiff guidewires. We then compared the cohort of 307 patients with a 304-patient back-to-back collective who retrospectively received conventional transjugular pacing for TAVI. This control group was retrospectively gathered by analyzing all TAVI patients between 1 June 2019 and 17 February 2020, prior to the COVID lockdown in Germany. We therefore assume that both our cohorts represent standard TAVI collectives.

TAVI was performed by cardiologists in a hybrid catheterization laboratory in the presence of both cardiac surgeons and anesthesiologists under conscious sedation by an experienced operator team of four interventionalists following a standardized procedure protocol in accordance with current guidelines. Aortic valve prostheses were implanted under fluoroscopic guidance via the femoral access route, with continuous radial blood pressure monitoring and cerebral protection when possible. Patients received either a balloon-expandable Edwards SAPIEN S3®, a Boston Lotus®, or a self-expanding Medtronic Evolut Pro+® TAVI prosthesis. The choice of implanted device type was made by at least two experienced interventionalists in accordance with applicable recommendations at the time of implantation. To reduce selection bias, no valves or patients were excluded, regardless of today's latest valve recommendations.

We analyzed various baseline factors, including left ventricular ejection fraction (LVEF) obtained via echocardiography, stage of decompensation (NYHA stage), occurrence of chest pain, and relevant comorbidities.

In addition, periprocedural factors, such as radiation exposure time, procedural duration, complications, both rhythmologic and non-rhythmologic, TAVI success, and short-term mortality, were considered. Adverse events were categorized into endpoints linked to wire pacing (need for transvenous pacing/failure of wire pacing) and other MACCEs. MACCEs were defined as permanent pacemaker implantation, all-cause in-hospital mortality, major bleeding, delirium, or stroke. All data were gathered from our SAP-based clinical information system. Data collected was approved by the local ethics committee (request numbers 283/21 and 178/14) and adhered to the ethical guidelines of the 1975 Declaration of Helsinki.

### Statistical analysis

Continuous variables are presented as mean ± SD and compared using a *t*-test or the Mann–Whitney *U*-test for unpaired comparisons. Categorical variables are presented as counts and percentages and were compared using the chi-square (*χ*²) test. Differences were considered statistically significant at *p* < 0.05. Statistical analyses were performed mainly using SPSS, version 20.0 (SPSS Inc., Chicago, IL and IBM, New York).

We compared the baseline characteristics and procedural parameters between patients in whom wire pacing was successful and those in whom it was unsuccessful and assumed that statistically different variables between groups could influence the success of wire pacing. Univariate binary logistic regression analysis uncovered significant predictors that might affect the efficacy of the wire pacing technique. Multivariate analysis was performed to validate the findings of the univariate analysis. All clinically relevant variables were included in the multivariate model, regardless of their significance in the univariate analysis. This approach was chosen to assess whether the variables identified as significant in the univariate analysis remained independent predictors after adjustment for other factors.

## Results

### Baseline characteristics

As shown in Table 1, the average age of patients in the wire pacing cohort was 80.9 ± 6.5 years, compared with 80.2 ± 6.3 years in the transjugular group (*p* = 0.15). Both groups included slightly more male individuals (55% in the wire pacing group vs. 53% in the transvenous pacing group, *p* = 0.6). The occurrence of coronary artery disease (CAD) was 67.1% in the wire pacing group and 55.6% in the transvenous group (*p* = 0.15). A history of prior cardiac operation was present in approximately one in nine patients (11.4%) in the wire pacing group and 8.2% in the transvenous group (*p* = 0.58). Hypertonia was documented in 80.8% of patients in the wire pacing group compared with 94% in the transvenous group (*p* < 0.001). Diabetes was present in almost one in three patients (32.9%) in the wire pacing group and 26.5% in the control group (*p* = 0.07). Dyspnea was reported by almost all patients (99.7%) in the wire pacing group. Among these, 13.9% experienced dyspnea with ordinary exercise (NYHA II), 72.3% experienced dyspnea with less than ordinary exercise (NYHA III), and 13.5% reported dyspnea at rest (NYHA IV). In the control group, dyspnea was present in 95.1% of cases. The NYHA stages differed in the control group, with 29.3% classified as NYHA II, 56.6% as NYHA III, and 9.2% as NYHA IV. When excluding patients classified as NYHA stage I, the wire pacing group exhibited a significantly higher burden of dyspnea (*p* = 0.02).

**Table 1:** Patient characteristics.

Parameter	Wire pacing	Transjugular pacing	Total	Level of significance
*N*	307	304	611	
Age (years)	80.9 ± 6.5	80.2 ± 6.3	80.6 ± 6.4	0.15
BMI (kg/m²)	26.6 ± 4.4	27.4 ± 5.1	27.0 ± 4.8	**0** **.** **03**
Male sex, *n* (%)	169 (55)	161 (53)	330 (54)	0.6
GFR (mL/h)	57.6 ± 20.1	58.6 ± 23.1	58.1 ± 21.5	0.55
Peripheral artery disease (PAD), *n* (%)	111 (36.2)	134 (40.1)	245 (38.0)	0.45
Known history of CAD, *n* (%)	206 (67.1)	168 (55.6)	374 (62.2)	0.15
Known history of cardiac operation, *n* (%)	35 (11.4)	25 (8.2)	60 (9.8)	0.58
Chronic lung disease, *n* (%)	53 (17.3)	82 (27.2)	135 (22.3)	**0**.**004**
Hypertonia, *n* (%)	248 (80.8)	284 (94)	532 (87.1)	**<0**.**001**
Diabetes mellitus, *n* (%)	101 (32.9)	80 (26.5)	181 (29.6)	0.07
NYHA stage				
1, *n* (%)	1 (0.3%)	15 (4.9)	16 (2.6)	**0**.**02**
2, *n* (%)	42 (13.9)	89 (29.3)	131 (21.4)	
3, *n* (%)	219 (72.3)	172 (56.6)	391 (64.0)	
4, *n* (%)	41 (13.5)	28 (9.2)	69 (11.3)	
EuroScore II (%)	6.1 ± 6.0	5.8 ± 6.0	6.0 ± 6.0	0.48
STS (%)	4.1 ± 2.9	3.5 ± 2.7	3.8 ± 2.8	**0**.**01**
N-terminal pro-brain natriuretic peptide (NT-pro BNP, pg/mL)	4,133.9 ± 6,923.9	4,452.7 ± 20,653.8	4,292.2 ± 15,737.2	0.8
High-sensitivity Troponin T (hsTnT, ng/L)	39.9 ± 41.2	54.9 ± 186.0	47.4 ± 123.3	0.17
Left ventricular ejection fraction (LVEF in %)	55.3 ± 12.9	50.1 ± 11.9	52.8 ± 12.5	**<0**.**001**
Aortic valve area (AVA, cm²)	0.8 ± 0.2	0.8 ± 0.2	0.8 ± 0.2	0.29
PGmax (mmHg)	67.1 ± 27.2	66.0 ± 23.6	66.6 ± 25.1	0.62
PGmean (mmHg)	40.3 ± 17.3	39.7 ± 15.2	40.0 ± 16.3	0.65
Systolic pulmonary artery pressure (SPAP, mmHg)	46.7 ± 15.4	41.0 ± 15.6	43.8 ± 15.6	0.60
ECG before TAVI				
Atrial fibrillation, *n* (%)	126 (41.0)	109 (35.9)	235 (38.5)	0.18
AV block I°, *n* (%)	71 (23.2)	76 (25.5)	147 (24.4)	0.58
Left anterior fascicular block, *n* (%)	25 (8.1)	23 (14.5)	48 (11.3)	**0**.**033**
Left bundle branch block (LBBB), *n* (%)	19 (6.2)	32 (10,5)	51 (8,4)	0.052
Right bundle branch block (RBBB), *n* (%)	34 (11.1)	37 (12.2)	71 (11.6)	0.67

Significant values (*p* = 0.05) are in bold.

As shown in Table 2, cardiac markers were elevated in both groups. NT-pro BNP levels were 4,133.9 ± 6,923.9 pg/mL in the wire pacing group and showed greater variability in the control group (4,452.7 ± 20,653.8 pg/mL, *p* = 0.8). Similarly, high-sensitivity Troponin T (hsTnT) levels were elevated in both groups (cutoff: 14 ng/L) and were not significantly different (39.9 ± 41.2 ng/L was the average in the wire pacing group vs. 54.9 ± 186.0 ng/L in the control group) (*p* = 0.17). Mean left ventricular ejection fraction was normal in the wire pacing group (55.3 ± 12.9%) and was significantly higher in the control group (50.1 ± 11.9%, *p* < 0.001). Severe aortic stenosis was present in both groups, with an aortic valve area of 0.8 ± 0.2 cm² in both the wire pacing and transjugular pacing groups (*p* = 0.29). In the wire pacing cohort, the maximal gradient measured was 67.1 ± 27.2 mmHg, with a mean gradient of 40.3 ± 17.3 mmHg, and the corresponding values in the control group were 66.0 ± 22.6 mmHg and 39.7 ± 15.2 mmHg, respectively (*p*=0.62 and 0.65), consistent with severe aortic stenosis. Most patients received Edwards SAPIEN S3 (52%) and Medtronic Evolut Pro+ (47.7%) TAVI valves.

**Table 2:** Procedural characteristics.

Parameter	Wire pacing	Transjugular pacing	Total	Level of significance
Duration of the procedure (min)	52.1 ± 15.9	59.0 ± 15.1	55.5 ± 15.5	**<0** **.** **001**
Radiation exposure time (min)	15.0 ± 5.3	20.8 ± 14.0	17.9 ± 9.1	**<0**.**001**
Use of Edwards SAPIEN valve, *n* (%)	161 (52.4)	59 (19.4)	220 (36)	
Use of Medtronic Evolut valve, *n* (%)	146 (47.6)	115 (37.8)	261 (42.7)	
Use of Boston Lotus valve *n*, %^a^	0 (0)	130 (42.8)	130 (21.3)	
Device success, *n* (%)	301 (98.0)	295 (97.4)	596 (97.7)	0.93
PGmax (mmHg)	20.0 ± 9.1	21.8 ± 10.7	20.9 ± 10.1	**0**.**03**
PGmean (mmHg)	11.0 ± 5.2	11.9 ± 6.1	11.4 ± 5.8	**0**.**03**
Significant aortic insufficiency, *n* (%)	6 (2.0%)	18 (6%)	24 (3.9)	**0**.**04**

^a^ The Boston Lotus program has been discontinued, and like many other valves, it is not commercially available anymore.

Significant values (*p* = 0.05) are in bold.

### Procedural data

The average procedural duration in the wire pacing group was 52.1 ± 15.9 min, including a radiation exposure time of 15.0 ± 5.3 min . The average procedural duration in the transvenous group was 59.0 ± 15.1 min, including a radiation exposure time of 20.8 ± 14.0 min. Not implanting a transvenous pacemaker therefore reduced the procedural duration and radiation exposure time significantly (both *p*-values < 0.001). Device success rates were approximately 98% in both groups (*p*-value = 0.93). The residual maximal and mean gradients after the TAVI procedure were 20.0 ± 9.1 and 11.0 ± 5.2 mmHg in the wire pacing group and 21.8 ± 10.7 and 11.9 ± 6.1 mmHg in the transjugular group, respectively. Regarding MACCE, the in-hospital mortality rate was 1.6% in the wire pacing group and 2% in the transjugular group (*p* = 0.74). Major bleeding events occurred in 1.3% of patients (Table 3) in the wire pacing group and in 4% of patients in the transvenous group (*p* = 0.025). Delirium was observed in 2.3% of cases in the wire pacing group and in 5.6% of patients in the control group (*p* = 0.035). Stroke/TIA occurred in 3.3% of patients in the wire pacing group and 4.3% in the transvenous pacing group (*p* = 0.51). Permanent pacemakers were implanted in 10.2% of patients in the wire pacing group and in 18.8% of cases in the transjugular group (*p* = 0.0023).

**Table 3:** Clinical course and relevant events.

Parameter	Wire pacing	Transjugular pacing	Total	Level of significance
Necessity for a permanent pacemaker, *n* (%)	31 (10.2)	57 (18.8)	88 (14.5)	**0** **.** **002**
Without Boston Lotus, *n* (%)		23 (13.2)		0.298
In-hospital death, *n* (%)	5 (1.6)	5 (2)	10 (1.8)	0.740
Major bleeding events, *n* (%)	4 (1.3)	13 (4)	17 (2.7)	**0**.**025**
Delirium, *n* (%)	7 (2.3)	17 (5.6)	24 (4)	**0**.**035**
Stroke, *n* (%)	10 (3.3)	13 (4.3)	23 (3.8)	0.510

Significant values (*p* = 0.05) are in bold.

### Wire pacing

Among all patients who underwent wire pacing during TAVI, sufficient pacing was achieved in 93.8% of cases (Table 4). This was assessed before valve implantation, and non-responsive patients received a transvenous pacemaker. The primary reason for wire pacing failure was insufficient capture of the electric signal by the guidewire (84.2%). In fewer cases (15.8%), patients experienced extrasystole triggered either by pacing or occurring intrinsically, all of which led to unwanted cardiac motion, i.e., a failure of wire pacing.

**Table 4:** Wire pacing.

Insufficient wire pacing, *n* (%), of which	19 (6.2)
Insufficient capture, *n* (%)	16 (84.2)
Extrasystole while pacing, *n* (%)	3 (15.8)
Periprocedural necessity for a pacemaker, *n* (%), of which	16 (5.4)
Emergency pacing by wire possible, *n* (%)	15 (97.7)

During the procedure, 5.4% of patients experienced a hemodynamically relevant atrioventricular block, necessitating placement of a transvenous pacemaker. Among these patients, all but one (93.8%) were stabilized using wire pacing until a transvenous pacemaker was introduced.

**Figure 1:**
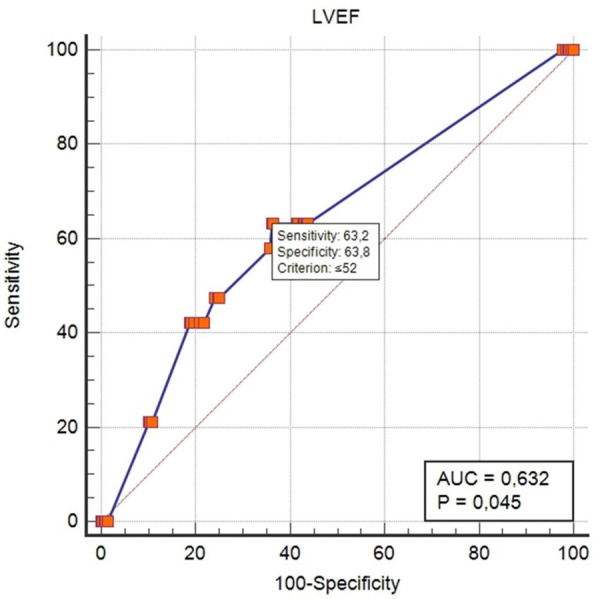
ROC curve analysis: prediction of insufficient pacing as a function of left ventricular ejection fraction (LVEF). AUC, area under the curve.

We observed most baseline and a few procedural parameters to analyze any influences on the success of wire pacing. While factors such as age, sex, valve type, or a known history of CAD were negative (Table 5), the left ventricular ejection fraction was the only parameter that showed a significant association, with an odds ratio of 0.965 and a *p*-value of 0.037. This finding is also confirmed in Table 6 in the multivariate analysis. The subsequent ROC analysis states a *J*-value of 0.632, reaching an LVEF of 52% (Figure 1).

**Table 5:** Predictors for insufficient wire pacing (logistic regression, univariate).

Variable	Odds ratio	Level of significance
Age	1.025	0.506
BMI	0.969	0.571
Sex	0.884	0.796
CAD	0.845	0.258
History of cardiac surgery	1.555	0.180
Lung disease	0.917	0.460
Diabetes mellitus	1.087	0.807
Atrial fibrillation	1.065	0.708
Left anterior fascicular block	0.611	0.616
AV block I°	0.590	0.388
Left bundle branch block		0.113
Right bundle branch block	0.429	0.361
Hypertonia	0.885	0.836
Systolic pulmonary artery pressure	0.996	0.839
Valve type (cat.)	n.m.	0.875
NYHA class (cat.)	n.m.	0.696
LVEF	**0** **.** **965**	**0**.**037**

Significant values (*p* = 0.05) are in bold.

**Table 6:** Predictors for insufficient wire pacing (logistic regression, univariate).

Variable	Odds ratio	Level of significance
Age	1.093	0.195
BMI	0.918	0.352
Sex	0.666	0.555
CAD	0.486	**0** **.** **028** ^a^
History of cardiac surgery	2.798	**0**.**023**^a^
Lung disease	0.812	0.198
Diabetes mellitus	2.007	0.207
Atrial fibrillation	0.912	0.722
Left anterior fascicular block	n.m.	0.998
AV block I°	0.308	0.128
Left bundle branch block	n.m	0.998
Right bundle branch block	n.m.	0.998
Hypertonia	2.712	0.269
sPAP	0.995	0.861
Valve model	1.109	0.880
NYHA	n.m	0.995
NYHA I	n.m	1.000
NYHA II	n.m	1.000
NYHA III	n.m	1.000
LVEF	0.948	**0**.**030**

^a^ CAD and history of cardiac surgery have a *p*-value lower than 0.05 because of overfitting; we therefore do not consider them as independent variables influencing wire pacing.

Significant values (*p* = 0.05) are in bold.

## Discussion

Wire pacing emerged from the need to stabilize the heart during TAVI procedures and has developed into a practical alternative to conventional temporary pacing. Today, it represents an important component of modern cardiological interventions, as it significantly shortens TAVI procedures and reduces radiation exposure time. In this analysis, we evaluated the safety and efficacy of wire pacing in patients with relevant symptomatic aortic valve stenosis and compared the outcome with those of a cohort of patients undergoing transjugular pacing.

Baseline parameters in which the transjugular group performed significantly worse included BMI (*p* = 0.03), incidence of lung disease (*p* = 0.004), incidence of hypertonia (*p* < 0.001), and mean LVEF (*p* < 0.001). Importantly, when comparing the NYHA stages of both groups, the transjugular group included fewer patients in NYHA stages II–IV (*p* = 0.002), suggesting that the significance does not influence the procedure.

The incidence of delirium was significantly lower. The lack of a pacemaker wire during the in-hospital stay (that could confuse older patients), as well as other elements of fast-track protocols, may have contributed to the reduction in periprocedural delirium.

An important discussion point influencing our results is the use of different TAVI valves. Edwards SAPIEN and Medtronic valves were used in a balanced manner in the wire pacing cohort, while the main valve type used in the transjugular group was the Boston Lotus due to historical practice preferences. As the Boston Lotus valve has been associated with higher rates of permanent pacemaker implantation (8), the increased incidence of permanent pacemaker implantation observed in the transjugular group (*p* = 0.0023) is likely caused by the different valves rather than the wire pacing technique itself. When comparing the procedures without Boston Lotus valves, there is no significant difference between the wire pacing and transjugular pacing groups (incidence 10.2% vs. 13.2%, *p* = 0.3). Although the Boston Lotus platform has since been discontinued, we did not censor the Boston Lotus from our study, as it represented a standard valve at the time of implantation, and we wanted to reduce selection bias.

We interpret the significance of the pressure gradient after the TAVI procedure (*p* = 0.03) in relation to the overall improvement of the procedure. After accounting for all these limitations, the parameters that cannot be factored out are the procedural duration (*p* < 0.001), radiation exposure time (*p* < 0.001), and the occurrence of major bleeding events (*p* = 0.03). We argue that the time, material, and radiation spared by avoiding transjugular pacemaker implantation can outweigh the risk of wire pacing failure, as a transvenous pacemaker is always on standby.

Within the wire pacing cohort of 307 patients, we consecutively implanted Edwards SAPIEN and Medtronic TAVI valves without the routine use of a transvenous pacemaker. In 16 cases, atrioventricular block occurred following TAVI implantation, making the placement of a transvenous pacemaker compulsory. Of these patients, 15 could be supported by wire pacing until a transvenous pacemaker could take over. In the long run, 31 patients required permanent pacemaker implantation.

**Figure 2:**
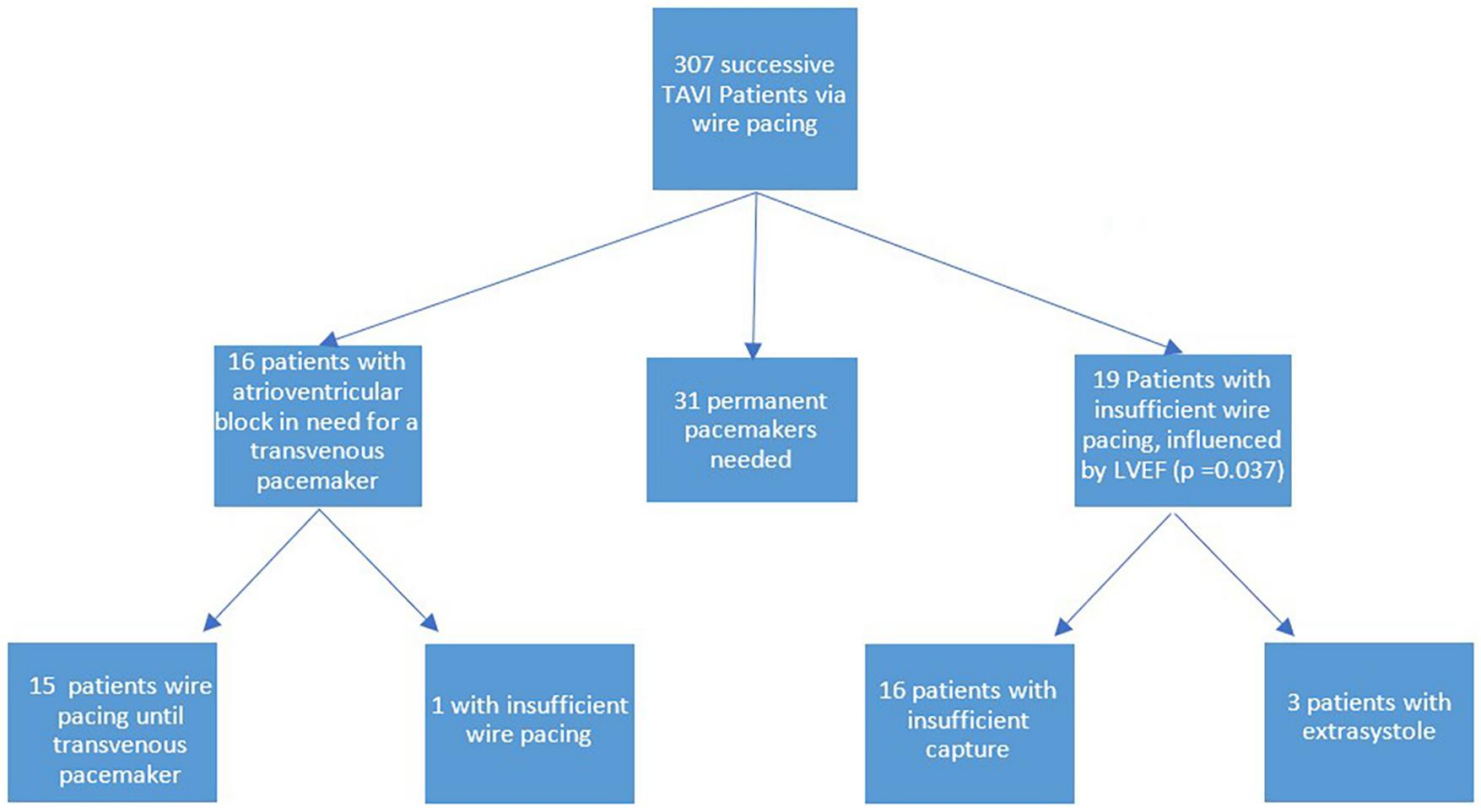
Flow chart: Wire pacing in TAVI.

In the 19 patients in whom wire pacing was insufficient, the most common reason was inadequate electrical capture, while the other cases were attributable to extrasystole negated cardiac immobilization. The only factor that can be used to predict the difficulty of wire pacing is the LVEF, with a cutoff value of 52%, which indicates that patients with heart failure should undergo wire pacing with more caution. These main findings are summarized in the graphical abstract (Figure 2).

We do not think that the wire type is relevant to the success of wire pacing. The wires used in wire pacing are sourced from different manufacturers; however, they are all extra-stiff wires that exhibit similar conductance properties. Literature only suggests minor differences in these guidewires (9).

It is obvious that cardiologists aim to offer the best possible safety net for their patients, who entrust them with their health. It might seem logical to perform TAVI with as many backup measures as possible. Until now, this has included the routine use of a transvenous pacemaker, not only to induce rapid pacing but also to rescue a patient in the event of a TAVI-associated AV block. Nonetheless, transvenous pacemakers can be complicated by pericardial effusion or pneumothorax. Their use is also associated with a higher radiation dose for both the cardiologist and the patient, as well as a longer procedural duration. An underestimated side effect in a cohort with multiple comorbidities is infection, especially involving the freshly implanted valve, which often cannot be traced back to transvenous pacemaker implantation. In fact, the incidence in TAVI patients has been estimated at approximately 2% within 4 years (10). As it is very difficult to quantify the incidence of pacemaker-linked systemic infections, the case of pacemaker lead infection highlights the seriousness of undetected infections.

For these reasons, with the growing safety net around procedures like TAVI, and as data and experience are gathered on how to make this procedure as comfortable as possible, we think that wire pacing is a viable and safe alternative that does not prevent escalation to a standby transvenous pacemaker, if necessary. This finding is consistent with the data from other studies on wire pacing (5, 6). Newer studies even go so far as to state that insecurities about the proper indication for a pacemaker lead to overtreatment, suggesting that the pacemaker risk in TAVI is overestimated (11) and that implantation should be more dependent on risk factors. For example, the presence of a right bundle branch block is an important predictor that has been consistently identified in multiple studies as an indicator of a higher risk of permanent pacemaker implantation (12). This is because TAVI causes a left bundle branch block in approximately 10% of cases (11). In our analysis, right bundle branch block did not alter the effectiveness of wire pacing.

While our data so far show that wire pacing itself is safe and highly effective, two important factors should be noted. First, successful left ventricular wire pacing requires the correct positioning of the wire within the ventricle. While all our patients were treated at a high-volume center by highly experienced interventionalists, a learning curve may exist for performing LV pacing during TAVI, which operators should be aware of. Second, this technique requires arterial access, meaning that it cannot be performed during the procedure. Consequently, in cases requiring longer pacing or pacing due to conduction disturbances that are present or threatening to occur, implantation of a transjugular pacemaker is needed. Therefore, patients at risk due to conditions such as the presence of a right bundle branch block before the procedure may not be suitable for such short-term wire pacing. Furthermore, all patients requiring permanent pacemaker implantation also require a transjugular temporary pacemaker after the procedure, as permanent pacemaker implantation should not be performed on the same day due to the possibility of conduction recovery.

As heart failure often comes with adverse cardiac remodeling and fibrosis, it explains why a lower ejection fraction is associated with a lower response to wire pacing. As this method has not been in use for a long time, further scientific advancements may lead to the development of next-generation arterial pacing devices that are not altered by cardiac ejection fraction.

### Limitations

We would like to point out that the nature of this study is mostly retrospective, as it involves a historical cohort treated with the Boston Lotus platform. While this platform has since been discontinued due to its association with higher rates of conductance disturbances, its inclusion allowed us to get unbiased insight into our data. Therefore, it was more important to have a consecutive TAVI cohort. To address this limitation, we performed a subgroup analysis excluding the Boston Lotus cohort, which confirmed the non-inferiority of wire pacing. We would like to emphasize that our study was not designed to demonstrate the superiority of wire pacing. The delay in time between the control group and the wire pacing cohort is due to the COVID-19 pandemic, which interrupted standard TAVI protocols.

We are well aware that a multivariate analysis has several limitations. Although we included all clinically relevant variables in this analysis, this approach is associated with a statistical risk of overfitting and multicollinearity and thus generally requires further validation. However, we refrained from additional modeling, as the results of the multivariate analysis confirmed what we observed in the univariate analysis, with LVEF remaining significant.

In addition, although LVEF was found to be a significant parameter that could limit the success of wire pacing, with an area under the curve of 0.632, it is not strongly correlated with wire pacing failure.

## Conclusion

As TAVI becomes increasingly accessible to younger patient cohorts, the implementation of fast-track protocols will play an ever more significant role. Within this setting and against all prior reservations, wire pacing has been established as a viable non-inferior alternative and a procedure with fewer complications than traditional transjugular venous pacing. Although a certain degree of caution is advised in patients with heart failure, this method is effective in saving significant time and resources. More importantly, it enables cardiologists to implant a TAVI without exposing patients to the risks and complications associated with a transvenous pacemaker, while maintaining relatively high security, as almost all rhythmological consequences can be handled, as shown in this study. We look forward to larger and comparative studies that may determine other relevant parameters to estimate the predictor-balanced risk of transvenous pacemakers compared to the benefits of wire pacing in the era of personalized medicine and bring TAVI to a broader cohort of low-risk patients.

## Data Availability

The data analyzed in this study are will be made available upon reasonable request. Requests to access these datasets should be directed to Sascha d'Almeida (lordayayi@yahoo.de).

## References

[1] Burgazlı KM Chasan R Kavukçu E Neuhof C Bilgin M Soydan N Transcatheter aortic valve implantation: our experience and review of the literature. Balkan Med J. (2012) 29(2):118–23. 10.5152/balkanmedj.2012.004 25206979 PMC4115866

[2] Webb JG Pasupati S Achtem L Thompson CR. Rapid pacing to facilitate transcatheter prosthetic heart valve implantation. Catheter Cardiovasc Interv. (2006) 68(2):199–204. 10.1002/ccd.20829 16810701

[3] Gupta S Annamalaisamy R Coupe M. Misplacement of temporary pacing wire into the left ventricle via an anomalous vein. Hellenic J Cardiol. (2010) 51(2):175–7. 20378522

[4] Oneglia C Simoncelli U. Brief report: 2D-echo diagnosis of interventricular septal perforation by a temporary pacing electrode. Minerva Cardioangiol. (1993) 41(12):603–5. 8139782

[5] Stąpór M Trębacz J Wiewiórka Ł Ostrowska-Kaim E Nawara-Skipirzepa J Sobczyński R Direct left ventricular wire pacing during transcatheter aortic valve implantation. Kardiol Pol. 2020 78(9):882–8. 10.33963/KP.15440 32567288

[6] Faurie B Souteyrand G Staat P Godin M Caussin C Van Belle E Left ventricular rapid pacing via the valve delivery guidewire in transcatheter aortic valve replacement. JACC Cardiovasc Interv. 2019 12(24):2449–59. 10.1016/j.jcin.2019.09.029 31857014

[7] Faurie B Abdellaoui M Wautot F Staat P Champagnac D Wintzer-Wehekind J Rapid pacing using the left ventricular guidewire: reviving an old technique to simplify BAV and TAVI procedures. Catheter Cardiovasc Interv. (2016) 88(6):988–93. 10.1002/ccd.26666 27510946

[8] Eksik A Yildirim A Gul M Aslan S Tosu AR Surgit O Comparison of Edwards Sapien XT versus lotus valve devices in terms of electrophysiological study parameters in patients undergoing TAVI. Pacing Clin Electrophysiol. (2016) 39(10):1132–40. 10.1111/pace.12917 27418419

[9] Tamura Y Tamura Y Konami Y Suzuyama H Horio E Yamada M Comparison of left ventricular pacing performance among pre-shaped guidewires designed for transfemoral-approach transcatheter aortic valve implantation. Heart Vessels. (2022) 37(3):460–6. 10.1007/s00380-021-01938-4 34524498

[10] Ando T Ashraf S Villablanca PA Telila TA Takagi H Grines CL Meta-analysis comparing the incidence of infective endocarditis following transcatheter aortic valve implantation versus surgical aortic valve replacement. Am J Cardiol. (2019) 123(5):827–32. 10.1016/j.amjcard.2018.11.031 30545481

[11] Mangieri A Montalto C Pagnesi M Lanzillo G Demir O Testa L TAVI and post procedural cardiac conduction abnormalities. Front Cardiovasc Med. (2018) 5:85. 10.3389/fcvm.2018.00085 30018969 PMC6038729

[12] Russo E Potenza DR Casella M Massaro R Russo G Braccio M Rate and predictors of permanent pacemaker implantation after transcatheter aortic valve implantation: current status. Curr Cardiol Rev. (2019) 15(3):205–18. 10.2174/1573403X15666181205105821 30516109 PMC6719385

